# Supplementary figures and images for: Gas concentration mapping and source localization for environmental monitoring through unmanned aerial systems using model-free reinforcement learning agents
Source: PLoS One. 2024 Feb 23;19(2):e0296969. doi: 10.1371/journal.pone.0296969 (PMC10889584; doi:10.1371/journal.pone.0296969)

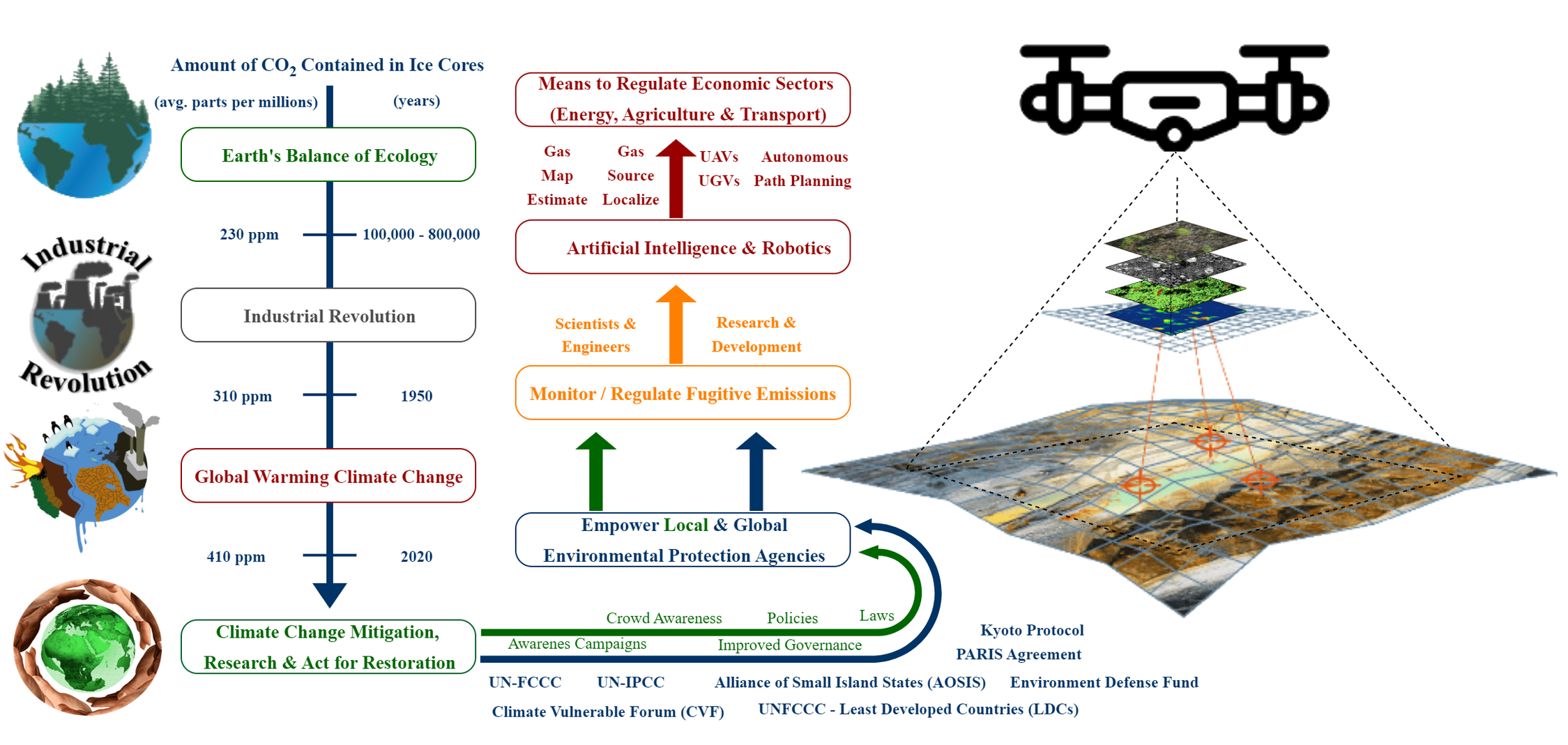

Supplement: S1 Graphical abstract — (TIF) [file pone.0296969.s002.tif]
