# Supplementary material for: Gas concentration mapping and source localization for environmental monitoring through unmanned aerial systems using model-free reinforcement learning agents
Source: PLoS One. 2024 Feb 23;19(2):e0296969. doi: 10.1371/journal.pone.0296969 (PMC10889584; doi:10.1371/journal.pone.0296969)
Supplement: Attachment — Submitted filename: Response to Reviewers.docx [file pone.0296969.s003.docx]

**Response to Reviewers**

*(****Ref 1*** *are for the “****Manuscript.docx****”)*

*(****Ref 2*** *are for the “****Revised Manuscripts with Track Changes.docx****”)*

**Response to Reviewer 1 Comments**

| **Sr** | **Comments** | **Response** | **Ref 1** | **Ref 2** |
| --- | --- | --- | --- | --- |
| 1 | The abstract is too long. Some information could be inserted in the introduction chapter. | The abstract has been reduced to 192 words. A few lines have been moved under the Introduction section. | Page: 2,  Line: 27-40 | Page: 2,  Line: 30-52 |
| 2 | This manuscript is not a project to have a challenges chapter or a goal one. This usually are included in the first chapter, meaning the Introduction. The presentation and the discuss of the findings must be concisely ( i.e., the goal was already presented in the abstract). | The goal subsection has been omitted as directed by the reviewer.  The challenges subsection has already been placed under the introduction section. | Page: 4-5,  Line: 83-110 | Page: 5-6,  Line: 103-134 |
| 3 | The contribution is usually specified after the presentation of the measurement method and the results. | A brief section under the name of “work contributions’ has been added just before the conclusion section. | Page: 32,  Line: 573-572 | Page: 36-35,  Line: 650-669 |
| 4 | The chapter called “Reinforcement learning and gas concentration mapping” should be revised. | The chapter has been improved by adding brief detail about the Q-learning agents.  The algorithm through which the Q-table is updated has also been added under section “Trajectories Generation” | Page 18-19,  Line: 331-338, 343-353  Page: 24,  Line: 457-467 | Page: 21-22,  Line: 404-411, 415-427  Page: 27-28,  Line: 540-550 |
| 5 | Table 4 should not be inserted in the text. | We suggest that Table 4 should be allowed in the text because Table 5 picks the failed and successful scenarios from the Table 4. | NA | NA |
| 6 | It is not clear. The test was done in a controlled environment. What about the real conditions? | This has been briefed under “data acquisition” section.  The data was collected in real conditions while autonomous path generation was done in simulation. The simulation results were validated from the experimental data. | Page: 14,  Line: 247-257 | Page: 16,  Line: 303-313 |
| 7 | The challenges that you mentioned in introduction how will influence your determinations? How many failed data will be then? | The challenges if not met, would drastically influence the results. The impacts of failing to meet challenges are briefly presented at the end of each challenge. | Page: 4-5, Line: 86-87, 92-94, 96-101, 105-107 | Page: 5-6, Line: 108-109, 114-115, 118-126, 128-130 |
| 8 | In my opinion you should revised your manuscript to be an interesting one for the readers. Not a boring one. | To engage the reader’s interest, the following changes have been made:  a). A table of comparison with relatable works has been presented a comparison  b). Two additional diagrams: i). updating the Q-table and ii). drone path generation, have been added.  c). Repetitions of contexts have been omitted  d). The tone of speech of the article has been improved.  (c and d can be viewed in track changes) | Page: 11-13, Table: 1  Page: 24, Figure: 10  Page: 24, Figure: 11 | Page: 13-15, Table: 1  Page: 28, Figure: 10  Page: 28, Figure: 11 |
| 9 | Briefly specify the present methods and their limits and yours new-one with benefits and limits. | Two brief sections by the name of “Work Contributions” and “Limitations’ have been added just before the conclusion.  To highlight the edge a table has been added that presents a comparison of the presented work with the most cited and recent works. | Page 11-13:  Table 1  Page: 32-34, Line: 553-586 | Page 13-15:  Table 1  Page: 36-37, Line: 650-683 |

**Response to Reviewer 2 Comments**

| **Sr** | **Comments** | **Response** | **Ref 1** | **Ref 2** |
| --- | --- | --- | --- | --- |
| 1 | Is this method applicable to all types of gas? | The answer is a ‘No’, as the dispersion behavior of gases vary because of their molecular masses and other factors. For this work, the RL agent was trained on a ‘heavier than air’ gas, butane (C_4_H_10_), hence, limiting the scope of this work to heavier gases.  This has also been added under the “Limitations” section. | Page: 33-34, Line: 573-587 | Page: 37-38, Line: 670-683 |
| 2 | Is there any limitation to apply this approach? | A brief section by the name of “Limitations’ has been added before the conclusion. | Page: 33-34, Line: 573-587 | Page: 37-38, Line: 670-683 |

**Due Adherence to the Journal Requirements from the Editor’s Office**

| **Sr** | **Comments** | **Response** | **Ref 1** | **Ref 2** |
| --- | --- | --- | --- | --- |
| 1 | Ensuring The PLOS ONE’s style requirements | PLOS ONE style sheet checked from the PDFs. | NA | NA |
| 2 | The grant information in the ‘Funding Information’ and ‘Financial Disclosure’ sections do not match  *(*When you resubmit, please ensure that you provide the correct grant numbers for the awards you received for your study in the ‘Funding Information’ section.)* | The grant information has been added into the “Funding Information” section. | NA | NA |
| 3 | Funding information should not appear in the Acknowledgments section or other areas of your manuscript.  *(*Only the information present in the Funding Statement section of the online submission form will be published)* | Funding information in the acknowledgement has been removed. | Page: 1 | Page: 1 |
| 4 | Remove any funding-related text from the manuscript.  To inform the editorial staff for updating the Funding Statement.  *(*Currently, your Funding Statement reads as follows:*  *“The funders had no role in study design, data collection and analysis, decision to publish, or preparation of the manuscript.”)* | It is ensured that manuscript does not contain any funding information. | NA | NA |
| 5 | To include the amended statements within the cover letter; the staff will change the online submission form on the author’s behalf. | The statement has been modified under the funding information step; it is requested to the editorial staff to update the information on author’s behalf. | NA | NA |

**Adapting to the Recommendations from the Editor’s Office**

| **Sr** | **Comments** | **Response** | **Ref 1** | **Ref 2** |
| --- | --- | --- | --- | --- |
| 1 | Significant text overlap between submission and previous work in the [introduction, conclusion, etc.] | The introduction and conclusion sections have been in terms of texts and tone. The changes can be seen in tracked changed version. | Page: 2-5, Line: 46-48, 55-57, 79-82  Page: 38-37, Line: 587-591, 600-602, 606-611 | Page: 3-6, Line: 59-62, 70-74, 100-103, 136-142  Page: 38-37, Line: 685-695, 705-709, 715-719 |
| 2 | Revise the manuscript to rephrase the duplicated text | The overall language and text have been improved and can be seen through track changes. | NA | NA |
| 3 | Provide details as to how the current manuscript advances on previous work | To highlight the edge of this works, the subsection under related work “Use of Reinforcement Learning for path planning” has been extended. A table has been added that presents a comparison of the presented work with the most cited and recent works. | Page: 11-13:  Table 1 | Page: 13-15:  Table 1 |
| 4 | Review the reference list to ensure that it is complete and correct | Reference list had been updated with the addition of some new references. | Page: 35-37 | Page: 39-41 |
| 5 | Any changes to the reference list should be mentioned in the rebuttal letter that accompanies your revised manuscript | Reference list had been updated with the addition of some new references. | Page: 35-37 | Page: 39-41 |
