# Supplementary material for: Gas concentration mapping and source localization for environmental monitoring through unmanned aerial systems using model-free reinforcement learning agents
Source: PLoS One. 2024 Feb 23;19(2):e0296969. doi: 10.1371/journal.pone.0296969 (PMC10889584; doi:10.1371/journal.pone.0296969)
Supplement: Attachment — Submitted filename: Response to Reviewers.docx [file pone.0296969.s004.docx]

**Response to the Comments from Reviewer**

*(****Ref 1*** *are for the “****Manuscript.docx****”)*

*(****Ref 2*** *are for the “****Revised Manuscripts with Track Changes.docx****”)*

| **Sr** | **Comments** | **Response** | **Ref 1** | **Ref 2** |
| --- | --- | --- | --- | --- |
| 1 | A discussion on sensor type is needed. The reason behind that is the efficiency and selectivity of butane sensors is highly dependent on sensor types. Was it a high temperature sensor or a low temperature sensor? What was the response time of this sensor for a reliable reading? What was the calibration procedure? what will be impact of temperature variations on the performance of this sensor? | The following subsections have been added in the study for the needed discussion:   1. Sensor selection and specifications 2. Metal Oxide (MOS / MOX Sensors) 3. Advantages and limitations 4. Strategies incorporated to overcome limitations | Page: 16-21  Line: 303-394 | Page: 17-21  Line: 308-399 |
| 2 | Along the same lines, it will be nice to add whatever was not done from the above-mentioned steps in the limitations or future work sections. | This has been highlighted under subsection ‘factor of pressurized gas container.’  Limitation section has been updated as directed. | Page: 19  Line: 363-365  Page: 39  Line: 692-695 | Page:19  Line: 368-370  Page: 39  697-700 |
| 3 | Another important discussion to be added in the data acquisition section and/or limitations section is the impact of gas plumes emitted by a pressurized container. The gas temperature will be significantly different as it comes out of the pressure vessel. Hence the sensor readings may report a concentration value that is not a true indicative of a source location. Special adjustments will be required to calibrate the sensor array against this anomaly. | A specific subsection, “Factor of pressurized gas container” tries to address this comment alongside admission of the limitation for not considering the change in pressure of gas molecules on the identification gas source. | Page: 19  Line: 354-365 | Page: 19  Line: 371-383 |
| 4 | And finally include any and all of the above data sets to the submitted data. As an example, you should include the calibration factors of all sensors that you used. | The calibration factor has been addressed under “Strategies incorporated to overcome limitations”. | Page: 18-21  Line: 343-393 | Page: 19-20  Line: 348-398 |

**Response to the Comments from the Editor**

| **Sr** | **Comments** | **Response** | **Ref 1** | **Ref 2** |
| --- | --- | --- | --- | --- |
| 1 | Please improve the abstract by briefly mentioning the results achieved in a quantitative manner. | The abstract has been updated and now includes the results achieved from the experiments quantitatively. | Page: 2  Line: 40-47 | Page: 2  Line: 40-47 |
| 2 | Role of UAV in detection of gases mentioned on Line 69 could benefit from a reference 10.1109/ICCIS49240.2020.9257602. | The suggestion has been incorporated under the literature review section. | Page: 6  Line: 121-123 | Page: 6  Line: 121-123 |
| 3 | Similarly, autonomous behavior of a UAV on Line 74 can be mentioned with 10.1371/journal.pone.0282055. | The suggestion has been incorporated under the literature review section. | Page 6  Line: 134-139 | Page: 6-7  Line: 139-143 |
| 4 | Moreover, with a website reference, please include date accessed. | The website references have been reverified and date of accessed has been added. | Page: 40  724-725 | Page: 40  Line: 729-730 |
| 5 | In Table 5, scenario 1 (row 1), please make sure that the closing brackets] are consistent with opening brackets [. In the final version, please provide high resolution image files. | The inconsistencies in the use of brackets have been removed in Table 5. | Page: 32  Line: 616 | Page: 28  Line: 531 |
